# Evaluation of Alveolar Ridge Dimensions by Socket Preservation Therapy Using a Bone Graft and Platelet-Rich Fibrin: A Randomized Controlled Trial

**DOI:** 10.7759/cureus.60388

**Published:** 2024-05-15

**Authors:** Javeria Khan, Sumanya Bandi, Sowmya Gangineni, Sahithi Kummari, Daniel G Pradeep, Talluri Hinduja

**Affiliations:** 1 Department of Periodontics, Periodont Multispeciality Dental Clinic, Amravati, IND; 2 Department of Periodontics, Meghna Institute of Dental Sciences, Nizamabad, IND

**Keywords:** radiovisiographical, cbct, prf, dfdba, socket preservation

## Abstract

Background: Following the loss of a tooth, the new edentulous area of the ridge will undergo several adaptive modifications due to changes in function within and surrounding the socket. This bone resorption explains the need for socket preservation techniques in areas of esthetic concerns and functional demands. Demineralized freeze-dried bone allograft (DFDBA) possesses greater osteoinductive potential due to the exposure of bone morphogenetic protein (BMP-3​​​​​​)​and collagen fibrils and can be used efficiently in socket preservation techniques. DFDBA yields better results when combined with an autologous platelet concentrate, such as platelet-rich fibrin. Therefore, we formulated this randomized controlled clinical trial to assess the clinical and radiovisiographical outcomes of platelet-rich fibrin (PRF) and DFDBAs for extraction socket preservation in humans at different time intervals.

Materials and methods: This was a randomized controlled trial with 100 people as study subjects, and they were randomly divided into two groups: the test group (DFDBA and PRF placed in the extraction socket) and the control group (natural healing of the extraction socket). Clinical and radiographic evaluation using radiovisiography (RVG) was done at baseline, three-month, and six-month intervals. Cone-beam computed tomography (CBCT) was used at six months to determine the bone density in the test and control groups.

Results: When compared from baseline to six months, the percentage change in clinical and RVG measurements for the test group was 15.96% (11.9064 mm) and 16.77% (12.1840 mm), respectively, whereas for the control group, it was 46.09% (14.0396 mm) and 47.61% (14.5716 mm), thus indicating lesser bone resorption in the test group as opposed to the control group. CBCT values also showed greater bone density for the test group (682.3120 HU) than the control group (503.8336 HU).

Conclusion: This study demonstrates the advantages of DFDBA bone graft with PRF compared to natural healing in achieving socket preservation by maintaining the marginal and buccolingual bone levels.

## Introduction

In the field of dentistry, implanting replacement teeth is an established process. The basic requirement for prosthetic tooth replacement using implants is a sound and anatomically viable bone that can support the implants and future prostheses without causing pathological damage. Additionally, in the esthetic areas, the emergence profile of the implants is an important aspect to consider when planning prosthetic rehabilitation. Thus, for a prosthetically driven, fully functional implant that also provides esthetic requirements, we always require a minimum amount of sound bone that is anatomically and physiologically viable.

Following the loss of a tooth, the new edentulous area of the ridge will undergo several adaptive modifications due to changes in function within and surrounding the socket. The size of the ridge will shorten right after several tooth extractions and additional restorations with removable dentures, as is also often known in both horizontal and vertical dimensions [1,2]. Once the tooth is extracted, simultaneous interrelated intra-alveolar and extra-alveolar processes occur. Pietrokovski and Massler examined the extent of this shift. They published their findings in 1967 [3] and reported that, due to the tissue modeling, the palatal or lingual aspect of the ridge became a new site for the edentulous site's center. Their observations were corroborated by research findings presented by Schropp et al. in 2003 [4], who suggested that if a tooth is part of an alveolar process and has a horizontal width of 12 mm, the edentulous site will only measure 6 mm in width a year after the tooth extraction and healing process. There will be a loss of 2 mm from the lingual portion and 4 mm of tissue from the buccal area of the site throughout this 12-month period [5]. This bone resorption explains the need for socket preservation techniques in areas of esthetic concerns and functional demands. Additionally, the socket preservation treatment can lessen or completely eliminate the need for additional augmentation procedures by preventing eventual hard and soft tissue collapse; otherwise, there might be a delay in the logical prosthetic rehabilitation, which can also lead to increased patient morbidity.

To arrest or reverse the process of this inevitable resorption after tooth extraction, various osteoinductive and osteoconductive biomaterials can be used, including autogenous bone grafts, allografts, recombinant human bone morphogenetic protein (rh-BMP), and recombinant human platelet-derived growth factor (rh-PDGF) [6]. Bone allografts, derived from individuals of the same species, are commercially available in tissue banks. Demineralized freeze-dried bone allografts (DFDBAs) or freeze-dried bone allografts (FDBAs) are chemically treated to remove the infectiousness of the virus, along with strong acids. They can be easily packed into the extraction socket without provoking immunologic responses, and they also have better healing capabilities. Out of the two commercially available bone allografts, DFDBA possesses greater osteoinductive potential due to the exposure of bone morphogenetic protein (BMP-3) and collagen fibrils [7].

DFDBA yields better results when combined with an autologous platelet concentrate, which has abundant growth factors [8]. To achieve this, the most promising, inexpensive, and effective vehicle, agent, or material to date is platelet-rich fibrin (PRF) [9,10]. This autologous biomaterial is composed of a fibrin matrix containing a representative concentration of fibrin, fibronectin, vitronectin, and thrombospondin, as well as high concentrations of platelets, leukocytes, and growth factors such as vascular endothelial growth factor (VEGF), platelet-derived growth factor (PDGF), and transforming growth factor (TGF).To assess the regenerative effects of grafts placed in extraction sockets, the most ideal methods employed are histological analysis, cone-beam computed tomography (CBCT), two-dimensional radiographs, and clinical measurements. CBCT is by far the safest and most specific method to assess bone regeneration, as there is very minimal distortion of the images and exact reproduction of the volume and other dimensions of the hard tissues [11]. Thus, we formulated a randomized controlled clinical trial with the aim of determining the clinical and radiovisiographical (RVG) outcomes of demineralized bone allograft, freeze-dried, and PRF for extraction socket preservation in humans at different time intervals.

## Materials and methods

This study was a randomized controlled trial carried out on two groups, each consisting of 50 patients randomly selected from a sample of 100 participants. The test group and control group will be distributed using a computer-generated randomization table based on the specified inclusion and exclusion criteria. Teeth that were grossly decayed or deemed hopeless were selected for the study. The ethical approval was obtained from the ethical committee of New Horizon Dental College and Research Institute and issued with approval number NHDCRI/18/2331. The patients enrolled and included in the study were over the age of 18, in good general health, had provided written consent to participate in the study, had at least one tooth indicated for extraction, had declared their intention to receive an implant restoration for the non-restorable tooth, and were available for follow-up examination. Current smokers, pregnant/lactating females, and individuals with any systemic disorders were excluded from the study. Questions were asked about the menstrual cycle (puberty and menopause).

**Figure 1:**
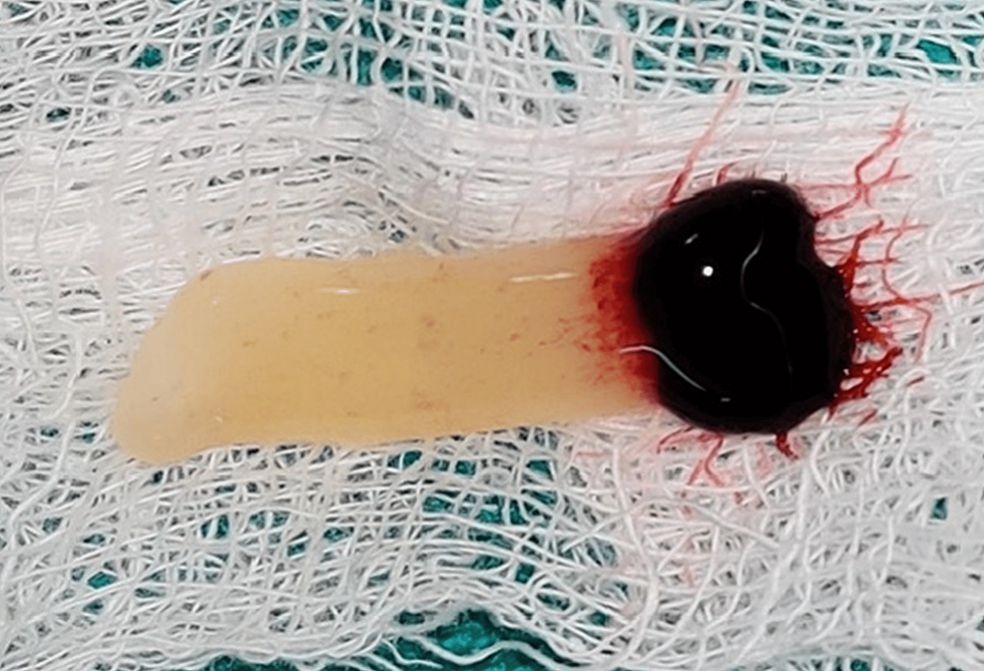
PRF prepared

The test group underwent medical and dental assessments (clinical measurements using a stent, RVG measurements, and CBCT used for measuring bone density were assessed at baseline after extraction and socket preservation), and an impression of the subject’s mouth was taken using alginate impression material and impression trays. Subsequently, a cast was fabricated. By scraping the tooth to be extracted on the cast, a stent was made with a fixed reference point constructed at the level of the marginal ridges of the adjacent teeth for recording the clinical measurements. Prior to tooth extraction, 5 ml of blood was collected from the patient's arm using yellow blood collection tubes. The blood was centrifuged using a universal centrifuge machine for 12.5 minutes at 2700 rpm to separate the PRF (Figure 1), according to Choukroun’s method [10].

**Figure 2:**
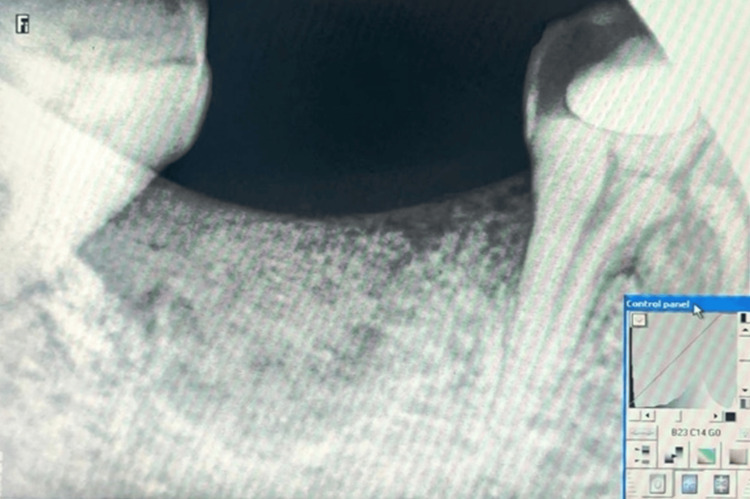
Radiograph after the placement of the graft

After administering local anesthesia (local anesthesia without adrenaline, approximately 2-3 ml, was injected for anesthesia; local infiltration was used for maxilla and nerve block was used for mandible subjects), a sulcular incision was made using a #15 blade around the indicated tooth (only molars, both upper and lower) for extraction. The tooth was extracted atraumatically using periotomes, thereby debriding the socket of any granulation tissue. Baseline measurements of socket walls were recorded clinically using a fixed reference point on the previously fabricated stent after being immersed in a disinfectant solution for 24 hours and rinsed with sterile water. The measurements were recorded using a HuFriedy UNC-15 periodontal probe (Chicago, Illinois). RVG measurements at baseline were recorded using the RVG software (Osirix 11.1 (Bernex, Switzerland) and Carestream 10.13 (Rochester, New York)) for assessment of bone density using CBCT. For statistical analysis, the IBM SPSS Statistics for Windows, Version 20 (Released 2011; IBM Corp., Armonk, New York) was employed, with the fixed reference point being the marginal ridge of adjacent teeth and the alveolar crest. As we used a stent with a ring at the level of occlusion of the adjacent teeth, if the stent could not be implanted at the six-month postoperative visit, subjects with this issue were excluded from the study. Only one case was reported with this issue; thus, we eliminated the factor of tooth tilt. A bone graft (Osseograft^TM^) was placed and packed in the extraction socket until it reached the gingival margin (Figure 2).

**Figure 3:**
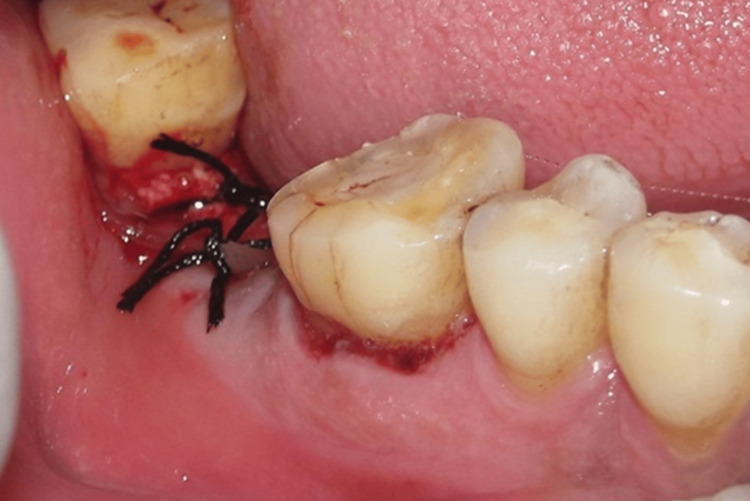
Suturing of the site

The extraction sockets were subsequently covered with PRF. The flaps were approximated by suturing them with a 3-0 non-absorbable surgical silk suture (Figure 3).

After providing the instructions regarding postoperative care, the patient was advised to use 0.12% chlorhexidine gluconate mouthwash twice a day, 24 hours after surgery. After 15 days, patients were called back for the removal of sutures. To evaluate wound healing, they were asked to return approximately three months after the baseline appointment to record the clinical and RVG measurements (Osirix 11.1 and Carestream 10.13) to assess bone density using CBCT. The IBM SPSS software was used for statistical analysis, as stated above. After six months, the patients were recalled to record the repeat clinical and RVG measurements.

Apart from these, CBCT measurements were also recorded on the same appointment to compare the bone density between the test and control groups. We used a negative control in our design and initially considered the null hypothesis before the alternative hypothesis. Implants were planned for all subjects; thus, those who did not employ regenerative materials required CBCT examination as a diagnostic technique. On CBCT (exposure time 5-40 seconds, radiation dose 1-15 mA at 90-120 kVp, field size 4x4 and 5x5), the bone density at the study area was measured in Hounsfield units (HU) [11]. Study treatment for the control group was the same as described above, except that the healing of the socket in this group took place naturally, and no attempts were made to preserve the socket.

The clinical operation was conducted by a surgeon who was blinded by the CBCT technique, and a biostatistician conducted the statistical analysis. As a result, all three individuals were blinded to the procedures performed by the others. All the values were tabulated, and the means were calculated for which descriptive and inscriptive statistics were applied. Paired and unpaired t-test was applied using the IBM SPSS software.

## Results

The data obtained from clinical, RVG, and CBCT measurements for 100 subjects in the test and control groups (50 subjects in each group) at baseline, three months, and six months were tabulated in an Excel sheet, and the means were calculated for all the parameters. These mean values were entered into the IBM SPSS software, and the results were obtained. The binomial test for gender returned a P-value of 1.0, and age showed no statistically significant difference. Table 1 shows the demographic data for both the test and control groups.

**Table 1:** Demographic data

Variables		Test	Control
Mean age (years)		38.73	42.96
Gender	Male	38	30
	Female	12	20

Tables 2, 3 show comparisons of clinical and RVG measurements at baseline, three-month, and six-month intervals in the test and control groups, respectively.

**Table 2:** Measurements for the test group *P-value of <0.05 is statistically significant RVG: radiovisiography; SD: standard deviation

Variables	Clinical measurements		RVG measurements	
Time interval	Mean value ± SD	P-value	Mean value ± SD	P-value
Baseline	10.2752 ± 0.89	0.000*	10.4352 ± 1.01	0.000*
3 months	11.1660 ± 0.80	0.000*	11.3884 ± 1.06	0.000*
6 months	11.9064 ± 0.75	0.000*	12.1840 ± 1.01	0.000*

**Table 3:** Measurements for the control group *P-value of <0.05 is statistically significant RVG: radiovisiography; SD: standard deviation

Variables	Clinical measurements		RVG measurements	
Time interval	Mean value ± SD	P-value	Mean value ± SD	P-value
Baseline	9.6060 ± 0.67	0.000*	9.8742 ± 0.69	0.000*
3 months	11.8798 ± 0.70	0.000*	12.3660 ± 1.09	0.000*
6 months	14.0396 ± 0.49	0.000*	14.5716 ± 0.93	0.000*

## Discussion

The process of socket preservation is used to maintain the soft and hard tissues of the alveolar ridge following extraction. Since the alveolar process depends on the tooth, bone loss after extraction happens very quickly in the first six months, resulting in a 40% decrease in alveolar height and a 60% reduction in alveolar width. Numerous studies have demonstrated that bone loss happens following extraction, with the labial side of the alveolar process experiencing greater bone loss than the lingual or palatal sides [2]. This resorption shifts the center of the ridge in a lingual/palatal direction.

Therefore, to assess the effects, we conducted a study using a randomized controlled methodology of a combination of DFDBA and PRF to achieve alveolar socket preservation as opposed to natural socket healing. A total of 100 subjects were selected for this study based on the inclusion and exclusion criteria. The demographic data of our study are shown in Table 1, wherein the mean age of the subjects in the test group was 38.73 years, and for the controls, it was 42.96 years. The test group had 38 males and 12 female subjects, whereas the control group had 30 males and 20 females. All the tooth extractions were carried out atraumatically using periotomes. In the test group, the DFDBA allograft (Osseograft) was filled and covered by PRF as a membrane after proper socket debridement. PRF was obtained according to Choukroun’s method, as explained earlier. In the control group, the sockets were debrided, and sutures were placed to allow them to heal naturally. Clinical measurements were taken at baseline, three months, and six months in the control and test groups. When these measurements at baseline were compared between the control and test groups, the mean was 10.0166 mm and 10.2752 mm, respectively. The difference in the mean was statistically insignificant (P-value = 0.103), thus denoting that at baseline, the alveolar socket walls were at the same height in both the test and control groups. Additionally, the RVG measurements at baseline were insignificant between the control and test groups (P-value = 0.344), as shown in Tables 2, 3. The patients were recalled at a three-month interval after the tooth extraction and the surgical procedure, at which time the clinical and RVG recordings were repeated. When clinical measurements were compared between the control and test groups, the mean heights were 11.8798 mm and 11.1660 mm, respectively, which was highly statistically significant (P-value = 0.000). Thus, less alveolar socket resorption occurred in the test group than in the control group. Similar results were obtained for RVG measurements at a three-month interval (P-value = 0.000).

Clinical measurements at six months and at the end of the study showed a mean of 14.0396 mm and 11.9064 mm for the control and test groups, respectively. Additionally, the RVG measurements showed a mean value of 14.0396 mm and 11.9064 mm for the control and test groups, respectively. When compared using inferential statistics, the results were highly statistically significant for both clinical and RVG measurements at six months (P-value = 0.000).

In our study, apart from clinical and RVG measurements, CBCT was also recorded at a six-month interval to compare the bone density in the control and test groups [11]. The bone density, as measured in HU, returned a mean value of 503.8336 HU and 682.3120 HU for the control and test groups, respectively, showing that relative to the control group, the test group had a higher value, which was statistically significant (P-value = 0.000).

This randomized controlled clinical trial was conducted to contrast the impacts of DFDBA and PRF as opposed to natural healing. It showed significant results in favor of using DFDBA with PRF as a membrane to achieve alveolar socket preservation for prosthetically driven implant placement. Baniasadi and Evrard (2017) [12], Quoc et al. (2018) [13], Hauser et al. (2013) [14], Anwandter et al. (2016) [15], Temmerman et al. (2016) [8], and Serafini et al. (2020) [16] corroborate the results of our study. However, Suttapreyasri and Leepong (2013) [17], Girish Kumar et al. (2018) [18], Zhang et al. (2018) [19], and Areewong et al. (2019) [20] showed insignificant results when PRF was used in their study for alveolar socket preservation. Thus, we would like to state that using PRF with any of the biomaterials as bone grafts for socket preservation could provide us with a predictable outcome if all the protocols and proper techniques that have been laid out are followed.

However, a better-designed longitudinal study, standardized and cost-effective protocols for fabricating PRF membrane, and non-antigenic bone grafting materials should have been used to assess the long-term effects of alveolar socket preservation. Better, standardized, and cost-effective imaging modalities could be used to evaluate the outcomes of alveolar socket preservation. Therefore, given the constraints of the research, we may state that socket preservation is a favorable and predictable method to avoid or delay alveolar bone resorption to achieve physiologically acceptable amounts of alveolar bone for the placement of prosthetically and esthetically functional osseointegrated implants.

The study has some limitations, such as the potential influence of age and gender on socket preservation and resulting differences in the final ridge dimensions. The hormone values of the subjects were not measured, and they need to be taken into account. CBCT was not used at the baseline of the study. In the future, alternative grafts can be explored to maintain the socket, increase the sample size, and include longitudinal follow-up in the study design.

## Conclusions

To conclude, this study demonstrates the advantages of DFDBA bone grafts with PRF compared to natural healing in achieving socket preservation by maintaining marginal and buccolingual bone levels. Contrary to the controls, the RVG methods also showed significant changes in the subjects who received bone grafts and PRF. Additionally, we discovered that, compared to those who did not receive any socket preservation, density levels are much higher in those who underwent socket preservation therapy.
